# Ketogenic and Modified Mediterranean Diet as a Tool to Counteract Neuroinflammation in Multiple Sclerosis: Nutritional Suggestions

**DOI:** 10.3390/nu14122384

**Published:** 2022-06-08

**Authors:** Danila Di Majo, Francesco Cacciabaudo, Giulia Accardi, Giuditta Gambino, Giuseppe Giglia, Giuseppe Ferraro, Giuseppina Candore, Pierangelo Sardo

**Affiliations:** 1 Department of Biomedicine Neuroscience and Advanced Diagnostics, Section of Human Physiology, School of Medicine, University of Palermo, 90127 Palermo, Italy; danila.dimajo@unipa.it (D.D.M.); giuditta.gambino@unipa.it (G.G.); giuseppe.giglia@unipa.it (G.G.); giuseppe.ferraro@unipa.it (G.F.); pierangelo.sardo@unipa.it (P.S.); 2 Department of Biomedicine Neuroscience and Advanced Diagnostics, Post-Graduate School of Nutrition and Food Science, School of Medicine, University of Palermo, 90127 Palermo, Italy; francesco.cacciabaudo@community.unipa.it; 3 Department of Biomedicine Neuroscience and Advanced Diagnostics, Section of General Pathology, School of Medicine, University of Palermo, 90134 Palermo, Italy; giuseppina.candore@unipa.it; 4 Euro Mediterranean Institute of Science and Technology-I.E.ME.S.T., 90139 Palermo, Italy

**Keywords:** Mediterranean, ketogenic, multiple sclerosis, diet, neuroinflammation, Tryptophan/Kynurenine ratio, brain derived neurotrophic factor

## Abstract

Ketogenic Diet is a nutritional pattern often used as dietotherapy in inflammatory diseases, including neurological disorders. Applied on epileptic children since 1920, in recent years it has been taken into account again as a tool to both reduce inflammatory burdens and ameliorate the nutritional status of patients affected by different pathologies. Multiple sclerosis (MS) is considered an immune-mediated neuro-inflammatory disease and diet is a possible factor in its pathogenesis. The aim of this work is to investigate the main potential targets of MS-related impairments, in particular the cognitive deficits, focusing on the alteration of biomarkers such as the Brain Derived-Neurotrophic Factor and the Tryptophan/Kynurenine ratio that could play a role on neuroprotection and thus on MS progression. Furthermore, we here propose nutritional suggestions which are useful in the development of a ketogenic diet protocol that takes advantage of the anti-inflammatory properties of low-carbohydrate foods from the Mediterranean diet to be applied to subjects with MS. In conclusion, this approach will allow one to develop the ketogenic diet combined with a modified Mediterranean diet as a possible tool to improve neuroinflammation in multiple sclerosis.

## 1. Introduction

Multiple sclerosis (MS) is a neurological disease characterized by an autoimmune response, inflammation, demyelination, gliosis, and neuronal loss [1]. It has a multifactorial nature and various environmental factors or metabolic conditions may have a role in its etiology [1]. Nutrition is now recognized as one of the possible risk factors for the development of MS and has potential applications in the management and treatment of the disease: eating habits and lifestyles can exacerbate or improve the symptoms of the disease, modulating the inflammation, interacting with human cells and the commensal gut microbiota [2].

In this context, the ability of dietary factors to interact with enzymes, transcription factors, and nuclear receptors of human cells is of striking importance [3,4]. Food can have medium and long-term effects on the production of circulating hormones which influence many physiological mechanisms responsible for the development of an inflammatory state and for an alteration of the redox cellular state [4,5]. Many MS patients are malnourished, clinically manifesting weight loss, overweight or obesity, as well as vitamin deficiency [6]. In patients with MS, malnutrition has been associated with impairment of the immune system, with negative impacts on cognitive function, inducing a chronic inflammatory state that increases the frequency of relapse and worsens the progression of the disease [7]. In addition, nutritional status negatively influences the effectiveness of drug therapy [8]. Several dietary strategies have been proposed over the years for the treatment of MS, complementary to drug therapy. Some of the models proposed appear particularly restrictive and difficult to implement over time, potentially leading to deficiencies in some nutrients. Despite the differences between the various diets, there are also some aspects in common, such as avoiding processed foods, foods with a high glycaemic index and foods rich in saturated fat; reducing the consumption of fatty red meat; and increasing the consumption of fruit and vegetables. However, clear evidence is lacking to support the benefit of any specific dietary regimen and well-designed, randomized controlled trials are needed [9]. The subject affected by MS often asks for a specific dietary regimen, but without guidelines, it could lead to malnutrition [6].

The Mediterranean diet (MeDi) in MS modulates the gut microbiota and low-grade inflammatory state. It has also recently been shown to reduce the risk of mental disorders, including depression and cognitive decline [10]. The influence of MeDi, rich in polyphenols, is reflected in Brain Derived Neurotrophic Factor (BDNF) levels, improving cognitive function, as shown in clinical trials [11]. This link becomes important if we consider the promising role of BDNF on neuroprotection demonstrated in experimental models of MS and clinical studies.

MeDi foods, especially vegetables, are rich in nutraceuticals. The use of nutraceuticals has been proposed for the enhancement of cognitive performances and reduction of neurodegenerative impairments, considering inflammation and oxidative stress as substantial factors that could induce neurological alterations [12,13,14]. Upon this view, nutraceuticals can find applications in neurodegenerative diseases such as MS.

Similarly, the application of the Ketogenic Diet (KD) protocol on mice with autoimmune encephalomyelitis (EAE) showed beneficial effects on disease progression, disability, cognitive status, and inflammatory markers by reducing the expression of enzymes involved in the biosynthesis of pro-inflammatory molecules [6]. A recent study has observed that the eight-week intervention with the very-low-calorie KD produced significant weight loss in obese patients, decreasing pro-inflammatory cytokine production, increasing adiponectin serum levels, and improving metabolic profile [15].

Hence, this evidence supports the interest in the development of a KD protocol modified and strengthened by the use of typical foods of the MeDi with lower concentrations of carbohydrates, in order to appreciate the beneficial properties of these two integrated dietary regimens on MS.

The aim of this review is to provide a comprehensive overview of the key points related to MS in order to pave the way to the application of a new dietary protocol that would be beneficial for the management of this neurological disorder. To this purpose, we have firstly investigate the main potential targets of MS-related impairments, in particular the cognitive deficits, focusing on the alteration of biomarkers such as BDNF and the Tryptophan/Kynurenine ratio whose neuroprotective metabolites can be modulated by nutritional approaches. Indeed, these biomarkers, that could play a role in neuroprotection and thus on MS progression, could be influenced by ketogenic and Mediterranean dietary protocols. Then, we focus on the consequences of malnutrition and its comorbidities on MS severity and progression. Finally, the potential anti-inflammatory properties of MeDi and KD on MS will be investigated, highlighting the effect of both protocols on BDNF production, and on inflammatory markers. In the light of all this evidence, nutritional suggestions based on the modified MeDi, and the KD will be proposed, comprising macro- and micro-nutrients that would be beneficial for the enhancement of MS patient compliance.

## 2. Multiple Sclerosis: Cognitive and Nutritional Factors

MS is a central nervous system (CNS) disease comprising macro and microscopic alterations: a focal inflammation resulting in macroscopic plaques and injury to the blood-brain barrier; and neurodegeneration with the microscopic impairment of axons, neurons, and synapses [1]. Macroscopically, myelin loss, edema, and axonal injury are encountered in MS plaques; whilst microscopical MS lesions strongly activate auto-immunity response recruiting macrophages, T and B cells, and initiating cytotoxic activities of microglia [16]. The MS disease course has been classified into seven categories, as in Lublin et al., 2014. The main four are the following: (1) relapsing-remitting (RR) is a very common disease course in which neurological symptoms develop over days to weeks and last 24 to 48 h; (2) primary progressive regards patients whose symptoms gradually deteriorate from the onset, without relapses; (3) secondary progressive shows an initial RR course followed by a slower neurologic deterioration; (4) progressive-relapsing typically shows a gradual deterioration with superimposed relapses. Furthermore, MS patients could present (5) a clinically isolated syndrome with a single episode of inflammatory CNS demyelination; (6) a fulminant syndrome with severe MS symptoms, multiple relapses and rapid progression; lastly (7) a benign clinical course with an overall mild disability.

### 2.1. The Role of BDNF in MS and the Influence of Dietary Factors on its Regulation

Once it was established that the impaired myelination process plays a crucial role in MS-induced neurodegeneration, the importance of neurotrophic growth factors in the myelin repair were given attention [17]. Among them, BDNF represents a gold standard in this context. Indeed, it is critical for neurodevelopment, for neuronal function and survival in the adult brain [18,19], but also for synaptic plasticity, especially in discrete brain regions such as the hippocampus, which is particularly important for behavioral and physiological processes [20,21]. It has been shown that BDNF contributes to neuroprotection via several effects, among which we encounter the impact on myelin integrity [22,23]. Several authors revealed that it can influence the distribution pattern of myelin structural proteins implicated in the integrity of the intact myelin sheath [24,25,26]. This myelin-protective effect helps guarantee mechanisms of myelin repair and eventually the degree of remyelination by downstream BDNF-induced endogenous pathways [27]. What has been unveiled is that it induces oligodendrocytes precursor proliferation, migration, and differentiation in the myelin damage foci [22,28], but also constitutes a survival factor for neurons by promoting remyelination of damaged axons [29].

Not surprisingly, recent research has explored its promising impact in the neuroprotection of MS in experimental models and clinical studies [24,25]. Indeed, some authors supported BDNF contribution to the remyelination of MS-induced lesions [26,30]. In particular, its levels resulted in decreased MS patients during RR phases, thus limiting the opportunity of individuals to repair myelin damage before a subsequent MS attack [31]. Whereas, BDNF serum levels are higher during the MS attack, though not sufficient to promote complete remyelination [26,32]. Collectively, these data could demonstrate its protective up-regulated activity to promote neuronal recovery in MS compared with healthy subjects and this could represent a reliable biomarker especially for MS diagnosis [33]. Even though some authors blame the putative discrepancy between serum and brain levels of BDNF [34], others indicate that plasma concentration reflects the brain altered levels in neurological disorders [35,36]. However, it is undeniable that the results obtained so far prompted novel pharmacological strategies in order to attempt the amelioration of MS disease by increasing endogenous or exogenous BDNF levels [37,38]. Intriguingly, its levels also seem to be implicated in cognitive deficits typical of neurodegenerative disorders such as AD, psychiatric disorders and MS [36]. Indeed, Hori et al., 2017 revealed that BDNF levels are strictly linked to the cognitive domains of memory and verbal learning, verbal fluency, and executive function. Moreover, its polymorphisms in MS have been correlated with cognitive performance and measures of brain atrophy [39,40].

In this context, the evaluation of BDNF biomarker in MS patients could be powerfully influenced by specific dietary protocols. Indeed, BDNF is implicated in glucidic homeostasis and energy balance [41], since an inverse correlation between blood glucose levels and BDNF release was found. Particularly, in rodent models, BDNF expression was correlated with glucose actions in the ventromedial hypothalamic nucleus. More intriguingly, KD was specifically implicated in the modification of plasma BDNF levels [42]. Indeed, beta-hydroxybutyrate (BHB) produced by this dietary protocol is able to cross the blood brain barrier (BBB) inducing the increase in the mitochondrial respiration and in turn NF-KB, that ultimately activates histone acetyltransferase p300/EP300 and consequently BDNF synthesis [42]. In addition to KD effects on BDNF levels, polyphenols present in the MeDi have been correlated to BDNF levels. It was outlined that the assumption of polyphenol-rich foods in MeDi activates nuclear factor CREB and thus BDNF levels, with positive outcomes on spatial memory performance in murine models. In addition, clinical trials pointed out that the consumption of high levels of polyphenols boosts cognitive function influencing neurogenesis via the specific activation of ERK/CREB/BDNF axis [11].

In the light of this evidence, KD and MeDi effects on BDNF levels could constitute a crucial turning point for supporting the cognitive relapse of MS through the control of specific dietary protocols.

### 2.2. Tryptophan-Kynurenine Metabolism in MS and the Influence of KD

Tryptophan (Trp) is an essential amino acid whose metabolites play key roles in several physiological processes; due to its very low reserves in the body, its deficiency rapidly manifests under various catabolic conditions. In a recent review its roles as a source of serotonin and melatonin, as a regulator of neurotransmission and its capability to influence circadian rhythm and cognitive functions have been highlighted and discussed [43]. It also influences the regulation of skeletal muscle mass and primarily influences immune responses. Trp has a stimulatory effect on proteosynthesis and its supplementation increases muscle mass and reduces adipose tissue. Trp blood levels decrease with age and inflammation: such decrease of Trp and the accumulation of its catabolite Kynurenine (Kyn) contribute to the development of sarcopenia. Kyn functions via numerous metabolic intermediates modulating inflammatory responses, oxidative stress and nicotinic and glutamatergic receptors [44]. In addition, Kyn serves important signaling functions in inter-organ communication and modulates endogenous inflammation. Several metabolites target the *N*-methyl-d-aspartate receptor as antagonists such as kynurenic acid (K-+A) or as agonists such as quinolinic acid (QA) and 3-hydroxykynurenine (3-HK), thus putatively exerting neuroprotective and neurotoxic effects on neuronal excitability [44,45].

It is possible to assume that in MS disease the indoleamine 2,3 dioxygenase (IDO) could influence TRP effects on immune functions since IDO can be activated during inflammation (stimulated by tumor necrosis factor-alpha (TNF-α), interferon (INF-α), etc.) to form Kyn, thus diminishing the amount of Trp available for the synthesis of serotonin, melatonin (increase in the Kyn/Trp ratio) and other important azoles. This leads to Trp depletion and consequently attenuation of proteosynthesis; such an effect could result in the rapid progression of muscle atrophy, sarcopenia, and polyneuromyopathy. Noteworthy, IDO was evidenced as a regulator of T cells’ response in MS’ clinical course via modulation of Th1/Th2 ratio. For instance, in RRMS patients’ IDO expression, that is augmented in relapsing phases, can be decreased by glucocorticoids administration together with its catalytic activity [46].

The effect of the Kyn/Trp ratio was evaluated on inflammatory states and neuronal excitability, highlighting that reductions in the Kyn/Trp ratio in favor of Trp through the consumption of tryptophan-rich foods improve skeletal muscle mass and ameliorate endogenous inflammation in MS patients [43]. The modulation of Trp–Kyn metabolism through lifestyle (diets, Branched-Chain Amino Acid (BCAA), aerobic exercise) could modify the balance in favor of Trp and its neuroprotective metabolites, ultimately supporting the treatment of MS disease with low grade chronic inflammation. Indeed, the KD protocol was found to downregulate the Kyn pathway in the hippocampus and at plasma level in rat models, revealing beneficial effects on neurodegenerative processes [47]. Lastly, BHB produced in the KD protocol determines a reduction of Kyn levels, an increase in KA and also in the KA/KyN ratio which sustains the neuroprotective of KD by inhibiting the kynurenina 3-monoxygenase enzyme that ultimately synthesizes KA [48].

### 2.3. Malnutrition in MS

Malnutrition is defined as an acute, subacute, or chronic state of overnutrition or undernutrition with or without inflammatory activity that leads to a change in body composition and functional variation. In patients with MS, malnutrition has been associated with impairment of the immune system. It affects mental function, respiratory muscle strength, and it contributes to exacerbate already existing symptoms, such as muscle wasting and weakness, fatigue and muscle spasm [49,50].

Many MS patients suffer from various forms of malnutrition, including weight loss, obesity or vitamin deficiency [6]. Malnutrition in MS is independent of the disease course and duration, number of attacks, disability status, and functional system involvement [49]. However, its incidence has not been well determined and there is a paucity of information regarding its functional consequences to MS patients. Epidemiological data show that the prevalence of chronic malnutrition was 11.8% in MS patients and only 2% in patients with other chronic disorders [49]. Our recent study of patients with RR-MS found that 70% of patients had a nutritional risk, of these 20% were undernourished and 50% were overnourished (unpublished data). Weight loss and cachexia are often present in patients with MS [51]. Evidence shows that there is a correlation between significant weight loss, cachexia, and a demyelinating lesion in the lateral hypothalamus [52]. Accordingly, a state of undernutrition can worsen the disease. When considering the condition of undernutrition in MS patients it is important to evaluate the impact of certain vitamin deficiencies: vitamin D deficiency has been widely reported, whereas folic acid and vitamin B12 deficiency has not. A recent review showed that vitamin D deficiency may be relevant to the development of the disease and to its severity [8,53]. In addition, the same authors also observed that low vitamin D levels also affect therapeutic response to medications. The above highlights the importance of vitamin D supplementation in MS patients in order to achieve plasma levels appropriate to the demands of the disease. Recently, it has been shown that vitamin D levels below 40 ng/mL are too low to keep the clinical condition under control and not to observe new lesions on MRI [8]. According to the literature [8], extensive controlled clinical studies would be necessary to establish standard levels of vitamin D supplements which are useful for patients with MS.

Overnutrition (defined as BMI > 24.9) includes overweight or obesity, that in infancy and adolescence can predispose to the onset of MS [54]. The Nurses’ Health Study I and II found that women with a BMI ≥ 30 kg/m^2^ at age 18 had a 2.25-fold increased risk of developing MS compared to those with a BMI in the normal range after adjusting for age, latitude, race/ethnicity, and smoking [55]. An epidemiological study on MS has estimated that eliminating childhood obesity could prevent approximately 15% of MS cases [56]. The condition of obesity and overweight in patients with MS increases the risk of comorbidities such as diabetes and cardiovascular diseases (CVDs) [57,58,59]. Both induce a chronic inflammatory state that worsens the disease, increasing the frequency of relapse with negative impact on cognitive function [7]. Obesity, overweight, and insulin resistance are interrelated components of the Metabolic Syndrome (MetS). Indeed, in a recent study on MS the onset of MetS was observed in 22% of patients and insulin resistance in 46% with a higher incidence in comparison with healthy controls [60,61]. Although the association between MetS or insulin resistance and degree of disability has not been unequivocally demonstrated, it has been observed that insulin resistance can exacerbate MS-related conditions as well as neurocognitive dysfunction and inflammatory and immune responses [62].

### 2.4. Role of Adipokines in the Pathogenesis of MS Associated with Obesity

Adipokines released from adipose tissue directly and indirectly control appetite, energy balance, immunity, angiogenesis, insulin sensitivity and lipid metabolism [63].The balance between proinflammatory (such as leptin) and anti-inflammatory (such as adiponectin-APN) mediators seems to play an important role in the pathogenesis of MS. Some studies have reported increased levels of leptin, resistin, and visfatin as well as decreased levels of APN in patients with RR-MS in comparison with healthy controls. Leptin and APN show an opposite role in the immune response. Leptin is a potent proinflammatory molecule. Its serum concentration increases in the active phase of RR-MS in untreated patients as compared to controls [64]. In the past, this adipokine has been considered a marker of disease activity and response to therapy but this was disproved in a 2018 randomized control trial [65,66]. The calorie restriction (CR) reduces leptin serum levels, by repairing the production of pro-inflammatory cytokines, demyelination, and axonal injury [59].

Otherwise, APN exhibits anti-inflammatory activity in immune system cells [67]; in fact, it inhibits the activation and proliferation of T and B lymphocytes and the phagocytic activity of macrophages, as well as the synthesis of both pro- and anti-inflammatory cytokines [68,69]. Reduced APN concentration was observed in patients with MS compared with healthy controls; interestingly, APN levels were higher in female patients than in male patients [70,71].

## 3. Mediterranean Diet

MeDi is associated with numerous health effects; it is one of the most varied and balanced diets and has been proven to be effective in terms of health protection, reducing the risk of CVD, diabetes mellitus and some types of diabetes mellitus and some types of cancer [72]. A recent cohort of Southern Italian patients have shown that the MeDi can have beneficial effects on MS long-term disability outcomes by positively modulating gut-microbiota and the low-grade chronic systemic inflammation, including CVD [10]. HELENA study has investigated the effects of MeDi on inflammatory state highlighting a counteracting effect of stress on inflammatory biomarkers with high MeDi adherence [73]. Finally, recent research has shown that a higher adherence to the MeDi is associated with a lower risk of mental disorders, including cognitive decline and depression [74]. Thus, the MeDi has a strong rationale for use in progressive MS [75].

### 3.1. Anti-Inflammatory Effect of MeDi

The Mediterranean dietary pattern is composed of fruit, vegetables, whole grains, nuts, seeds, legumes, and extra virgin olive oil (EVOO). Moreover, the traditional one is low in calorie intake as well as in animal proteins, especially in red and cured meat, with low amounts of saturated fatty acids and sugars and a high content of fibers [76]. It is a nutritional pattern and includes a lifestyle leading to successful aging and to a reduction in the onset of diseases and disabilities typical of the aging process [77,78]. The explanation could be found in the great variety of phytochemical compounds of its foods with proven nutraceutical properties [78]. These molecules showed antioxidant and anti-inflammatory effects, highlighted by reduction in serum levels of inflammatory mediators such as Creactive protein, Interleukin-(IL-)6, as well as many inflammatory biomarkers found in many chronic diseases, including MS [10,79,80]. Diet may influence the gut microbial composition and its metabolites that induce changes in progression and severity of MS disease. Additionally, many studies have demonstrated that MS patients exhibit intestinal dysbiosis with decreased abundance of *Clostridium*, Bacteroidetes and *Adlercreutzia* microbes [81]. Studies have shown that low-calorie diets comprising of high levels of fruits, vegetables and fish, which are typical in MeDi, promote beneficial gut microbiota and reduce inflammation in the body [82]. Gut commensal bacteria exert both pro- and anti-inflammatory responses by regulating T cell differentiation and immune responses in the gut [83].

The MeDi is rich in nutraceuticals, such as phenolic acids, flavonoids, stilbenes and lignans, terpenoids such as carotenoids and tocopherols, and unsaturated fatty acids. All foods containing these compounds can be considered as “functional foods”. Although a universal definition does not exist, the Functional Food Center defined them as “Natural or processed foods that contain known or unknown biologically active compounds; which, in defined, effective non-toxic amounts provide a clinically proven and documented health benefit for the prevention, management, or treatment of chronic diseases” [84]. Many studies have investigated how health status is directly affected by nutraceuticals, providing evidence that the increased intake of some nutraceuticals, above the habitual and recommended dose levels, can decrease both inflammatory status and reactive oxidative species [79]. In addition, it has been proposed that the use of nutraceuticals can improve cognitive performance and reduce neurological impairments considering inflammation and oxidative stress as substantial factors that could induce neurological alterations [85]. Upon this view, nutraceuticals can find applications in neurodegenerative diseases such as MS.

### 3.2. MeDi Foods Suggested for the Protocol to Be Developed

Foods of the MeDi that we will take into consideration for our proposed suggestions will be the ones with lower-carbohydrate concentrations that are richer in lipids, in order to enhance the anti-inflammatory properties of the MeDi with a modified approach. In particular, EVOO, the connecting food between Mediterranean countries, is the main source of fat in that diet and is a particularly rich source of phytochemical compounds, especially polyphenols. The main lipid constituents of olive oil are triglycerides. There are three main fatty acids in the triglyceride fraction: a monounsaturated fatty acid (MUFA), oleic acid (73.6%); a saturated fatty acid, palmitic acid (13.7%); and a polyunsaturated fatty acid (PUFA), linoleic acid (7.85%). The percentage ratio of fatty acids is 16.2%, 74.4% and 9.4% for SFA, MUFA and PUFA, respectively [86].

The remaining fraction contains about 230 bioactive molecules. These include lipophilic phenols (whose levels fall as olives grow up), sterols, color pigments (mainly chlorophylls and carotenoids), alcohols, waxes, aldehydes, esters, ketones, and phenolic compounds such as hydrophilic phenols [87,88]. EVOO might exert beneficial effects reducing the levels of markers of inflammation and conferring neuroprotection [88,89]. Hydroxytyrosol, oleuropein, and oleocanthal, polyphenols widely studied in vivo, in vitro and directly in human, inhibit Nuclear Factor-κB (NF-κB) pathway, showing ibuprofen-like activity and inhibiting cyclooxygenases 1 and 2, that are responsible for prostaglandin production [78,90,91]. Moreover, the phenolic, oleuropein aglycone inhibits TNF-α-induced matrix metalloproteinase 9 in a monocyte cell line with an interesting role in the development of inflammatory diseases [92]. In particular, a claim on the scavenging effect of EVOO compounds in radical species exists. A daily intake of 20 g of olive oil, which contains at least 5 mg of hydroxytyrosol and its derivatives (e.g., oleuropein and tyrosol) provides the expected beneficial effects. It relates to the impact of olive phenolic compounds on the protection of blood lipids from oxidative stress [93]. Nonetheless, it is to be considered that EVOO comes from olives. Although few studies exist on this food, it seems to exert anti-inflammatory and antioxidant effects, decreasing IL-6 and the levels of malondialdehyde, the main product of the PUFA peroxidation, and important index of oxidative stress [94]. Fish is another traditional MeDi food, although only in seaside countries and not in the countryside. The main components with anti-inflammatory properties are the omega-3 fatty acids, in particular eicosapentaenoic acid (EPA) and docosahexaenoic acid (DHA), especially in blue fish. Its meat seems less prone to induce pro-inflammatory cytokine production during digestion compared to red meat, due to the difference in lipid composition. Moreover, fish seems to modulate inflammatory cytokine production as well, with a possible positive effect on autoimmune disorders. In addition, stearidonic acid found in fish is a better precursor of α-linoleic acid for the synthesis of EPA whose effects on human health have been recognised for several years [95].

Of note, the benefits of fish consumption exceed the potential risks of heavy metal exposure [96,97]. Nuts and seeds are recognized as healthy food in MeDi, as well. They are rich in MUFA, PUFA, fibers, and omega-3. Almonds, pumpkin seeds, pistachio, and walnuts are frequently consumed by people of the Mediterranean basin, as a snack or in many recipes [98,99]. Herbs and spices, for example, parsley, oregano, rosemary, thyme, cinnamon, chili, and sage, are used daily in the Mediterranean area, both in the seaside and countryside villages. Their abundance in phytochemicals such as flavonoids, anthocyanins, isoflavones, terpenes, and isothiocyanates, confer the antioxidant and anti-inflammatory properties to these foods [100]. It is also noteworthy the caper. It is a plant that grows wildly in the Mediterranean basin that offers two edible options: the bud, the caper, and the fruit, the so-called “cucuncio”. In addition, in this case, anti-inflammatory and antioxidant properties were investigated, especially due to polyphenols [101]. In mice, caper fruit inhibits cytokine gene expression, including (IFN-γ, IL-17 and IL-4, probably due to saponins, flavonoids and alkaloids [102].

## 4. Ketogenic Diet

KD is a high-fat, low-carbohydrate diet that results in ketosis, elevations of fatty acids, serum levels, modulation of glycemia, and relative CR. In clinical practice, KD is an established treatment for drug-resistant epilepsy and the treatment of choice for Febrile Infection-Related Epilepsy Syndrome, a presumed inflammatory condition. KD may work by targeting “out of control” immune activation. There is a growing list of potential inflammatory pathway targets of KD including adenosine, ketone bodies, mechanistic target of rapamycin pathways, peroxisome proliferator-activated receptor-gamma (PPAR-γ), NLR Family Pyrin Domain Containing 3 (NLRP3) inflammasome, and gut microbiota [103]. KD is based on a drastic reduction in carbohydrates, associated with an increase in the proportion of proteins and fats. This condition pushes the body into ketosis, that is, into a metabolic state characterised by increased concentration of ketone bodies in the blood. Studies on mice with experimental EAE showed the beneficial effects of KDs on disease progression, disability, cognition and inflammatory markers, KDs could reduce the expression of enzymes involved in the biosynthesis of pro-inflammatory molecules [104,105].

### 4.1. Anti-Inflammatory Factors in the KD

Some features typical of KD could be responsible for its anti-inflammatory effects.

CR is a dietary restriction that drastically reduces energy intake without malnutrition [106]. It can modulate some inflammatory regulators such as NF-κ B inhibitor alpha (Nfkbia), tissue inhibitor of metalloproteinases-3 TNF-α, IL-6, COX-2, iNOS, VCAM-1, and ICAM-1 [107,108]. Thus, CR can regulate inflammation reducing the level of many pro-inflammatory mediators and pathways.

Omega-3 (n-3) and the omega-6 (n-6) are the two main groups of PUFAs. In nutrition, we recognize three types of omega-3 fatty acids in foods, which are alpha-linolenic acid (ALA), eicosapentaenoic acid (EPA), and docosahexaenoic acid (DHA). ALA consists of 18 carbon atoms while EPA and DHA are considered long carbon chains. These are essential fatty acids that we can only introduce through diet. ALA is a precursor of EPA and DHA but is capable of producing quantities of less than 15%. To increase the share of EPA and DHA, we can introduce them through diet. ALA is present in vegetable oils while EPA and DHA are represented in fish coming from the microalgae on which fish feed. DHA plays an important role as a component of cell membranes and is present in high concentrations in the retina and brain. EPA, on the other hand, plays an important role in the synthesis of eicosanoids and competes with arachidonic acid to produce prostaglandins (PG), thromboxanes (TX), and leukotrienes (LT). Higher concentrations of EPA than arachidonic acid drive the synthesis of eicosanoids with less inflammatory activity. Moreover, n-3 PUFA indirectly regulates transcription factors involved in the expression of inflammatory genes. In addition, n-3 PUFA might influence the composition of gut microbiota, enhancing the production of anti-inflammatory compounds [109]. n-6 PUFAs are found in a variety of animal products and in vegetable oils, such as canola and corn oil. They produce pro-inflammatory effects and due to their sources, the Western diet provides an excess of n-6 PUFAs, compared to n-3. Thus, it can be considered pro-inflammatory. Not enough data are available to establish the recommended dose of omega 3, the IOM (National Academy of Medicine) has been able, however, to establish the adequate intake in a healthy population based on age (Institute of Medicine, Food and Nutrition Board). Dietary reference intakes for energy, carbohydrate, fiber, fat, fatty acids, cholesterol, protein, and amino acids (macronutrients). Washington, DC: National Academy Press; 2005). According to the European Food and Drug Authority, the total daily intake of DHA and EPA should not exceed 5 g per day [110]. Dietary reference intakes for energy, carbohydrate, fibre, fat, fatty acids, cholesterol, protein, and amino acids (macronutrients). Washington, DC: National Academy Press; 2005). The adequate values are 1.6 g of ALA per day for males (age > 14 years) and 1.1 g of ALA per day for females (age > 14 years)[110]. The KD increases the levels of specific PUFAs that can bind and activate the considered potential anticonvulsant drug targets [111].

The KD’s anti-inflammatory properties could also be due to the variation of adenosine levels, as this molecule has anti-inflammatory activity [112]. It was demonstrated that adenosine can reduce systemic inflammation by modulating LPS-induced transmigration of polymorphonuclear cells and lowering pro-inflammatory mediators, such as TNF-α, IL-6, and CXCL2/3 [113]. Astrocytic adenosine kinase (ADK) can phosphorilate adenosine and lead it to clearance from the extracellular space. KD is able to lower the expression of ADK, consequently raising extracellular levels of adenosine and triggering the activation of inhibitory adenosine A1 receptor (A1AR). Most important, the effect of the KD on adenosine was associated with a decrease in electrographic seizure activity [114]. Both A1ARs and the adenosine A2 receptor are involved in inflammation, thus providing an opportunity for pharmacological intervention [115].

Ketone bodies, BHB and Acetoacetic Acid (AcAc), exert their neuroprotective role through the reduction of oxidative stress and ROS production by enhancing NADH oxidation and by inhibiting mitochondrial permeability transition. Ketone bodies seem to target inflammatory signaling cascade both by direct action on the inflammasome and ROS reduction [116]. Recently, the relevant role of microbiome in KD bioactive effects was also demonstrated. It can act in the antiseizure effect of the KD, using both an acute seizure model and an epilepsy mouse model. The gut microbiota transplantation after KD confers seizure protection in mice fed with a standard diet, with an increase of the GABA in the hippocampus [117].

### 4.2. KD and Neuroinflammation: The Role of NLRP3 Inflammasome

BHB, represents another anti-inflammatory mediator produced following the KD. It reduces nucleotide-binding oligomerization domain leucine-rich repetition and NLRP3 inflammasome-mediated activation of IL-1β [118]. This could be one of the reasons to explain the anti-inflammatory effects of the KD.

It exerts antidepressant-like effects, possibly by inhibiting NLRP3-induced neuro-inflammation in the hippocampus [119].

NLRP3 is a pattern recognition receptor of the innate immunity, belonging to the NOD-like receptor (NLR) subfamily. Together with the adaptor ASC protein PYCARD, it forms a protein complex able to activate caspase-1 and, consequently IL-1β, initially translated as pro-IL-1β. The inflammasome dysregulation has been implicated in different autoimmune diseases [120]. Recent studies show that the NLRP3 acts as a bridge between the innate and adaptive immune responses in the initial stages of MS and EAE by promoting the migration of macrophages, dendritic cells and myelin-specific autoreactive CD4^+^ T cells to the CNS [121,122]. Therefore, it can be considered a critical factor in the development of neuro-inflammation and an interesting therapeutic target in immune-related disorders [123].

## 5. Nutritional Suggestions to Develop a Dietary Protocol for MS Patients

Studies on mice with EAE showed beneficial effects of KD on disease progression, disability, cognition and inflammatory markers. The ketone bodies produced by KD are an alternative energy source for the brain and they are useful to reduce neuroinflammation possibly by inhibiting NLRP3. In addition, it seems ketone bodies are able to stimulate mitochondrial biogenesis and to reduce NLRP3 permeability by improving redox balance.

This section provides nutritional suggestions for developing a diet plan based on the KD that includes many foods from the MeDi, which are rich in polyphenolic compounds, to be applied to patients with MS type RR during the remission phases.

The nutritional plan, that could be developed, could have anti-inflammatory, immunomodulatory, and neuroprotective properties that could bring improvements on neuroinflammation and the redox state of the MS patient with benefits on the progression and course of the disease. Assuming that dietary intervention can (i) modulate the inflammatory state; (ii) protect against neurodegeneration; or (iii) promote nervous system repair [124], the choice of nutrients and their proportions is critical. This section pays particular attention to the importance for the MS patient to introduce macronutrients in the diet in the appropriate ratios. The effects of diet on MS may be the consequence of the direct action of metabolites produced by food or the effect of metabolites synthesized by the gut microflora or even diet-mediated changes in gut microbial composition.

The nutrition plan should include a classical KD protocol which is modified in some aspects. It could involve two steps: (1) an adaption phase and (2) a maintenance phase. The adaptation phase is divided into two periods: the first will last four weeks during which patients will be instructed to limit the intake of carbohydrate to 20 g/day, in order to establish the ketosis condition. In the second period of the maintenance phase, lasting one month, the patient will be asked to increase carbohydrate intake by 5 g each week until the maximum level of 40 g per day is reached. The carbohydrates used will be with glycemic index and glycemic load below 50 and six respectively to maintain an adequate state of ketosis and constant levels of blood sugar and insulin.

**Figure 1:**
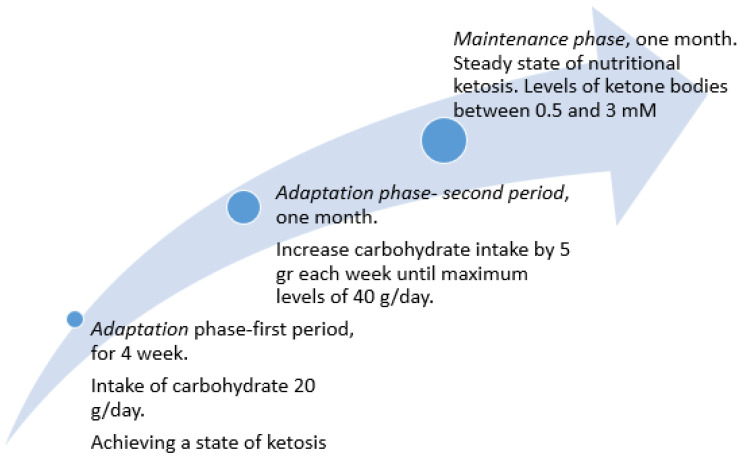
Trend of daily carbohydrate intake in the protocol to be developed to achieve proper levels of ketosis.

The maintenance phase lasts one month and is characterized by a constant state of nutritional ketosis (Figure 1). The ketone body levels will be maintained between 0.5 and 3 mM.

A similar nutritional approach was applied in a clinical trial and achieved good compliance in patients with MS. In this study the authors concluded that the KD diet is a safe and inexpensive complementary treatment option for MS. The implementation of the protocol is based on the international guidelines of ketogenic protocols [125], providing a caloric deficit that can vary from 300 to 500 Kcal depending on the BMI. It is also expected to have a daily intake of water equal to 0.4 mL per kg of body weight.

### 5.1. Macronutrients

Macronutrients will be divided into approximately 5–10% carbohydrates; 15–20% proteins; 70–80% fats (compared to a traditional KD characterized by 90% fat, 6% protein, and 4% carbohydrates).

Considering the effects of KD on the state of ketosis, on the lipid profile, and on the glycemic profile, taking into account the protective effect of BHB on neuroinflammation previously mentioned, it becomes important to pay attention to the fatty acid composition.

The percentage of SFA and PUFA in the KD influences ketosis status, lipid and glycemic profile. Short-term administration of a KD richer in PUFAs (15% SFA, 25% MUFA, 60% PUFA) induces a greater level of ketosis and improves insulin sensitivity without negatively affecting total or LDL cholesterol levels compared with a traditional KD high in saturated fats (60% SFA, 25% MUFA, 15% PUFA) [125]. A prospective study in pediatric MS has suggested that the increase in energy intake from saturated fat tripled the risk of relapse rate [126]. On the contrary, animal studies have found that PUFAs, especially alpha linolenic acid (ALA) and EPA and DHA have a beneficial effects on EAE by reducing the production of inflammatory cytokines and inducing PPAR in CNS infiltrating T cells [125,127]. In addition to immunomodulatory effects, PUFA’s prevent demyelination and promote neuroprotection and remyelination [124]. For the reasons outlined above, the protocol suggested will provide high concentrations of PUFAs with a qualitative distribution of fatty acids as follows: 15% SFA, 25% MUFA, 60% PUFA, the ratio between omega-3 and omega-6 will be 1:4 [128].

In a diet, the intake of proteins must be strictly controlled and animal proteins must be limited because of their proven pro-inflammatory effects, precisely for this reason Hernández et al. proposed a ratio between vegetable and animal proteins of 70 to 30. Data in the literature show that increased consumption of plant protein may be associated with a reduced risk of CVDs [129], type 2 diabetes, and inflammation [130,131]. Recently in the eighth edition of the Dietary Guidelines for American it was suggested to shift the ratio towards proteins of vegetable origin [132]. Regarding the amount of protein to be administered, it was demonstrated that a slightly higher-protein diet was able to both positively influence body weight regulation and reduce insulin resistance. This aspect becomes important if we consider that insulin resistance as well as other parameters of the MetS, which affects 30% of MS patients, have negative effects on the progression of the disease [130].

In addition, a recent meta-analysis of 32 randomized controlled trials showed a long-term positive effect of higher-protein diets on body weight management, which in turn could lead to lower glycated hemoglobin (HbA1c) [133]. Another recent study showed that a protein score with a higher protein energy percentage (E%) within the acceptable macronutrient distribution range for protein in combination with a higher plant to animal protein ratio, would be associated with a lower HbA1c level [134].

The daily protein intake provided should be between 0.9 and 1.2 g/Kg of body weight and will be characterized by a high ratio of plant to animal protein.

**Figure 2:**
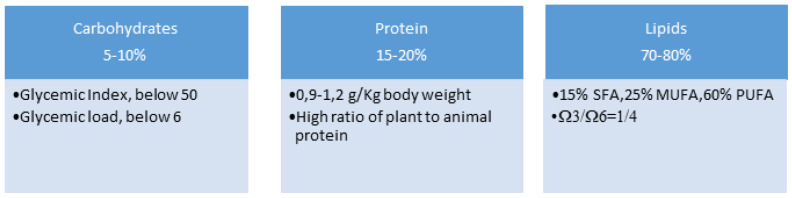
Qualitative and quantitative daily distribution of macronutrients into the future protocol proposed to be applied to patients with MS.

In brief, Figure 2 shows the qualitative and quantitative nutritional distribution suggested for the protocol to be developed.

When selecting foods for inclusion into the future protocol, particular attention will be paid to those rich in tryptophan in order to increase the Trp/Kyn ratio.

Therefore, taking into consideration that the recommended daily intake for an adult is estimated to be around 250–425 mg, corresponding to 3.5–6.0 mg/Kg/day, foods included in the modified MeDi and that we suggest to include in the protocol are: eggs which have an average content of (197 mg/100 g) and in particular the yolk which has a content of 237 mg/100 g; fish, with particular reference to sea bass (249 mg/100 g), tuna (237 mg/100 g), sea bream (259 mg/100 g), sardine (250 mg/100 g), sole (220 mg/100 g). Among meats, chicken (240 mg/100 g) and turkey rump (333 mg/100 g). Other recommended foods include almonds (394 mg/100 g), pine nuts (300 mg/100 g), cashews (240 mg/100 g), flaxseed (298 mg/100 g), sesame seeds, pumpkin seeds (Tabella di composizione degli alimenti, aggiornamento 2000, CEd EDRA). In our modified MeDi food suggestions, dark chocolate can also be added, despite not being part of the typical MeDi protocol, because it is particularly rich in Trp. The aforementioned foods will supplement those in Table 1 so that you could have a synergistic effect with the ketogenic diet on the antioxidant and anti-inflammatory properties.

### 5.2. Bowel Dysfunction in MS and Fiber Nutritional Suggestions

The protocol will need to pay special attention to the contribution of fiber. A patient with MS has a high prevalence of intestinal dysfunction whose origin is multifactorial, in fact it may depend on neurological alterations, polypharmacy, behavioral problems or motor skills. Constipation and fecal incontinence can coexist and alternate, impacting the patient’s quality of life and social interactions [139,140]. The percentage of patients with neurological bowel dysfunction varies from 39 to 73% and the bowel symptoms seem to be correlated to the Expanded Disability Status Scale (EDSS) and disease duration, but not with the type of MS [141]. However, it is not said that patients with a mild degree of malignancy do not present intestinal disorders; on the contrary, it seems that constipation may be a symptom which MS manifests itself with [142]. The regulation of stool consistency is important and can be modulated by the intake of both water and fiber. The management of the fiber amount (and laxative as well) is essential because on the one hand they need the formation of soft stools, preventing incontinence and constipation. On the other hand, the excess of both can cause bloating, in the presence of pan-gut dysmotility. Scant evidence exists on the use of laxatives, mostly from studies of neurological conditions or idiopathic bowel symptoms.

KDs are typically low in fiber which is needed not only for healthful intestinal function but also for the microbial production of beneficial colonic short-chain fatty acids [143], which enhance nutrient absorption, stimulate the release of satiety hormones, improve immune function, and have anti-inflammatory and anti-carcinogenic effects [144]. It has been suggested that the supplementation of KD with fiber and non-digestible carbohydrates might be advisable although data to confirm that supplementation could counteract the effects of very-low-carbohydrate diets on the gut microbiota are lacking [145].

Given the difficulty of achieving the appropriate amount of fiber through a KD protocol and the intestinal dysfunction that can be seen in the MS patient, it is planned to supplement our suggestions with natural substances with a laxative function to help intestinal function.

Laxative agents that can be considered include: (i) psyllium that is a bulking agent which might work for slow-transit constipation as shown in patients with Parkinson’s disease (PD) [146]; (ii) polyethylene glycol equally acts as an osmotic agent but does not rely on bacterial fermentation for its activation. The advantage is that it can be quite effective, and the dose can be adjusted within a wide range according to the patient’s need. In patients with PD, it has been proven to relieve constipation [147]; (iii) a stimulant laxative such as bisacodyl has been shown to be very effective in patients with chronic idiopathic constipation [148].

According to recent work, these laxatives may be effective in MS patients [139].

### 5.3. Micronutrients

A critical aspect of KD that must be kept under control regards micronutrients, vitamins and minerals which can easily become defective. Extreme carbohydrate restriction can profoundly affect diet quality, typically curtailing or eliminating fruits, vegetables, whole grains, and legumes and increasing the consumption of animal products. Very-low-carbohydrate diets may lack vitamins, minerals, fiber, and phytochemicals found in fruits, vegetables, and whole grains [149]. Low-carbohydrate diets are often low in thiamin, folate, vitamin A, vitamin E, vitamin B6, calcium, magnesium, iron, and potassium [150]. Physiologically, it has been shown that the deficiency of certain vitamins and minerals affects energy production and results in physical and mental fatigue and impaired cognitive functions, consequences that aggravate the clinical symptoms of MS [151].

Other suggestions include strict control of the intake of vitamins and minerals playing a key role in controlling inflammation and whose deficiency is related to a worse prognosis of the disease [151]. Given the deficiency of some micronutrients associated with KDs, in Table 2 are listed the micronutrients that will be added in the form of supplements in the recommended daily amounts.

## 6. Conclusions

The present review explored putative biomarkers implicated in MS-related alterations such as the role of BDNF and the Tryptophan/Kynurein ratio on cognitive deficits and eventual neuroprotection. Furthermore, we described the effects on the immune system, neuroinflammation and redox balance of both the modified MeDi and the KD regimen in order to develop a potential dietary protocol that could be applied to MS patients. We think that this nutritional approach may exert an enhanced effect compared to the individual dietary regimens on which it is based. The encouraging preclinical data on KD in MS disease and the result of the recent clinical trial on KD conducted in MS patients confirm and support our idea.

The application of the protocol and the possible confirmation of its effectiveness could be useful to formulate guidelines on proper nutrition for MS patients.

## Figures and Tables

**Table 1:** Foods to be included in the nutritional suggestions and their nutraceutical compounds with anti-inflammatory and antioxidant properties.

Food	Main Anti-Inflammatory/Antioxidant Components	Reference
Pistachio nut	Proanthocyanidins	[99]
EVOO/olive	Polyphenols (e.g., oleocanthal, hydroxytyrosol, tyrosol, oleuropein).	[88]
Almonds	Vitamin E, MUFA, PUFA	[88,98]
Capers	Phenolic compounds (e.g., rutin), tocopherols (e.g., gamma-tocopherol), carotenoids (e.g., lutein and beta-carotene), vitamin C.	[101]
Cinnamon	E-cinnamaldehyde, o-methoxycinnamaldehyde	[135]
Turmeric	Curcumin	[136,137]
Green tea	Epigallocatechin 3-gallate (EGCC)	[138]

Legend: EVOO: extra virgin olive oil. MUFA: monounsaturated fatty acid. PUFA: polyunsaturated fatty acid.

**Table 2:** Suggestions of micronutrients to include in the protocol, amounts that have shown effects on fatigue and cognitive function and recommended intake and tolerance levels for a healthy population.

Micronutrients	Effect of Supplementation with Vitamins and Minerals on Mental Fatigue and Cognitive Functions	RDA, AI, or UL in Healthy Subjects According to LARN
Vitamin D	Dose > 100 µg/die Effect = toxicity [152,153] Serum concentrations >150 ng/mL Effect = clinical condition of vitamin D toxicity characterized by hypercalcemia and hypercalciuria) [154]	RDA = 15 µg/die (adult 18–74 yo) RDA = 20 µg/die (>74 yo) UL = 100 µg/die [155]
Thiamine (B1)	Dose = 50 mg/die for 2 months (120 young women) Effect = improvement of attention threshold and mood [156]	RDA = 1.2 mg/die (men > 18 yo) RDA = 1.1 mg/die (women > 18 years) UL not defined [155]
Niacin (B3)	Dose = 250 mg/die Effect = modulation of NIACR1 expression on peripheral immune cells by improving sleep spectrum disorders in Parkinson’s disease [157]	RDA = 18 mg/die UL (Nicotinamide) = 10 mg/die UL (Nicotinic acid) = 900 mg/die [155]
Vitamin C	Dose > 2000 mg/die Effect = diarrhea or kidney damage [152,153]	RDA = 105 mg/die (men > 18 yo) RDA = 85 mg/die (women > 18 yo) UL not defined [155]
Pyridoxine (B6)	Dose > 1000 mg/die Effect = might mimic MS symptoms Dose < 50 mg/die Effect = nervous symptoms [152,153]	RDA = 1.3 mg/die (18–29 years) RDA = 1.7 mg/die (men) RDA = 1.5 mg/die (women, 60–74 yo) UL = 25 mg/die [155]
Vitamin E	Dose > 1500 IU/die Effect = possible toxicity	AI = 13 mg α-TE (men > 18 yo) AI = 12 mg α-TE (women > 18 yo) UL = 300 mg α-TE [155]
Folic acid (B9)	Dose= 1000IU of of alpha-tocopherol twice daily for three years Effect= No significant effect cognitive function, speed of processing, clinical global impression, functional performance, adverse events, or mortality [158] Dose = 800 µg/day for 3 years Effect = improvements of global cognitive functions, information-processing speed and memory storage [159] Dose = 400 µg/day for 2 years (+100 µg/day of B12) Effect = improvement of cognitive functions particularly long- and short-term memory [160]	RDA = 400 µg/die UL = 1000 µg/die [155]
Vitamin B12	Dose = 400 µg/day for 2 years (180 subjects with mild cognitive impairment) Effect = improved cognitive performance (full scale and verbal intelligence, memory) [160]	RDA = 2,4 µg/die (men and women) UL not defined [155]
Calcium		RDA = 1000 mg/die (18–59 yo) RDA = 1200 mg/die(≥60 yo) UL = 2500 mg/die [155]
Zinc	Dose = 15.30 mg/die (387 healthy adults 55–87 yo) Effects = better on spatial working memory [161]	RDA = 12 mg/die (men) RDA = 9 mg/die (women) UL = 25 mg/die [155]
Iron	Dose = 60 mg/die for 4 months (149 iron-deficient American women) Effect = 5-fold improvement in cognitive performance [162]	RDA = 10 mg/die (men ≥ 18 yo) RDA = 18 mg/die (women 18–49 yo) RDA = 10 mg/die (women > 50 yo) [155]
Magnesium	Dose = 20 mg/day Effect = reduced risk of depression [163]	AI = 2.7 mg/die (men > 18 yo) AI = 2.3 mg/die (adult women > 18 yo) UL not defined [155]

Legend. RDA, Recommended Daily Allowance; UL, Tolerable Upper intake Level; AI, Adequate Intake; LARN, Nutrients and Energy for Italian population; yo, years old; NIACR1, Niacin receptor 1; α-TE, Tocopherol Equivalent.
